# The Recent Epidemic of Measles in Buffalo

**Published:** 1900-08

**Authors:** F. C. Gram

**Affiliations:** Buffalo, N. Y.; Registrar of Vital Statistics, 460 Glenwood Avenue


### THE RECENT EPIDEMIC OF MEASLES IN BUFFALO.

By F. C. GRAM, M. D., Buffalo, N. Y.
Registrar of Vital Statistics.

MEASLES, as a rule, does not inspire much fear in the average person and is therefore no more dreaded than a severe cold. The fact that it is "only measles" is usually sufficient to put all precaution to sleep, which slumber continues until local quarantine measures prevent children from attending school, or until the undertaker calls, when the remainder of the family wake up.

Fortunately, the fatality from measles is very small, but no matter how small, some one may be the victim, and no one cares to have it a member of his household, if preventive measures will apply.

In examining the mortality statistics for Buffalo for some years we find that the deaths from measles for each year beginning with 1887, were respectively: 60, 35, 5, 56, 47, 18, 17, 11 and 99. In 1896, there were 342 cases with 33 deaths, in 1897 there were 976 cases with 38 deaths; in 1898 there were 46 cases with no deaths, and in 1899 there were 3,178 cases with 50 deaths.

As no reports of cases were kept in the Health Department, prior to 1896, it is impossible to say how many cases existed in the previous years for which the mortality rate only is given. We are, likewise, unable to say how the ratio of deaths corresponds to the number of cases, compared with the recent epidemic, but if it was anything similar there must have been fair-sized epidemics in 1887, 1890, 1891 and 1895.

This shows an intervening period of from three to five years between each outbreak of measles, of more than ordinary severity.

The year 1898 was a proverbial "lull before the storm," for during this year there were only 46 cases reported in the entire city, with not a single death.

Then came the great measles harvest. I have made a detailed table of cases and deaths for each month, from January, 1899, to May, 1900, as follows:

---

1. Read at the Semi-annual meeting of the Medical Society of the County of Erie, June 12, 1900.

### Cases of Measles by Wards, 1899–1900.

| Wards. | January. | February. | March. | April. | May. | June. | July. | August. | September. | October. | November. | December. | Total. | January. | February. | March. | April. | May. | Total. |
|---|---|---|---|---|---|---|---|---|---|---|---|---|---|---|---|---|---|---|---|
| 1 | ..... | ..... | ..... | ..... | ..... | ..... | ..... | ..... | ..... | ..... | 1 | 9 | 10 | 12 | 17 | 3 | 13 | 2 | 47 |
| 2 | ..... | 1 | ..... | ..... | ..... | ..... | ..... | ..... | ..... | 1 | 1 | 5 | 8 | 37 | 56 | 44 | 17 | 2 | 156 |
| 3 | ..... | ..... | ..... | 8 | 6 | 1 | ..... | ..... | ..... | ..... | 11 | 28 | 54 | 33 | 58 | 20 | 4 | 1 | 116 |
| 4 | ..... | 1 | ..... | ..... | ..... | 1 | 1 | ..... | ..... | 1 | 26 | 41 | 71 | 21 | 26 | 23 | 9 | ..... | 79 |
| 5 | ..... | ..... | ..... | ..... | ..... | 2 | 3 | ..... | ..... | ..... | 40 | 118 | 163 | 85 | 150 | 44 | 10 | 12 | 301 |
| 6 | ..... | ..... | ..... | ..... | ..... | ..... | ..... | ..... | ..... | ..... | 17 | 16 | 33 | 32 | 25 | 7 | 4 | 2 | 70 |
| 7 | ..... | ..... | ..... | 1 | 5 | ..... | 4 | 1 | ..... | 5 | 38 | 22 | 76 | 11 | 8 | 6 | 7 | ..... | 32 |
| 8 | ..... | 1 | ..... | ..... | 2 | ..... | ..... | ..... | ..... | 11 | 53 | 48 | 115 | 23 | 6 | 13 | 1 | 2 | 45 |
| 9 | ..... | ..... | ..... | ..... | ..... | ..... | 3 | 1 | ..... | 18 | 55 | 33 | 110 | 20 | 2 | 2 | ..... | ..... | 24 |
| 10 | ..... | ..... | 10 | ..... | ..... | ..... | 3 | ..... | ..... | 11 | 48 | 31 | 94 | 13 | 7 | 1 | ..... | 2 | 23 |
| 11 | ..... | ..... | ..... | 1 | 1 | ..... | ..... | 1 | 1 | 7 | 67 | 126 | 204 | 64 | 40 | 7 | ..... | 4 | 115 |
| 12 | ..... | ..... | ..... | ..... | 1 | 4 | ..... | ..... | 1 | 3 | 48 | 83 | 140 | 6 | 3 | 1 | 7 | 4 | 21 |
| 13 | ..... | ..... | ..... | ..... | 2 | 1 | 3 | ..... | 1 | 7 | 46 | 39 | 99 | 19 | 7 | 6 | 6 | 1 | 39 |
| 14 | ..... | ..... | ..... | 2 | 1 | ..... | ..... | ..... | 3 | 17 | 136 | 134 | 293 | 41 | 6 | 2 | 2 | ..... | 51 |
| 15 | ..... | ..... | ..... | 1 | 1 | 5 | 5 | ..... | ..... | 1 | 30 | 104 | 147 | 24 | 12 | 6 | 5 | 1 | 48 |
| 16 | ..... | ..... | ..... | ..... | ..... | 8 | 1 | ..... | ..... | ..... | 25 | 118 | 152 | 15 | 12 | 3 | 2 | 1 | 33 |
| 17 | ..... | ..... | ..... | 1 | 67 | 15 | 11 | ..... | 1 | 1 | 60 | 134 | 290 | 67 | 30 | 15 | 8 | 10 | 130 |
| 18 | ..... | ..... | 2 | 2 | 8 | 10 | 7 | 1 | 2 | 24 | 135 | 295 | 486 | 37 | 17 | 9 | 4 | 3 | 70 |
| 19 | 1 | 6 | 4 | 6 | 4 | ..... | 1 | ..... | ..... | 2 | 3 | 3 | 30 | 11 | 23 | 58 | 27 | 10 | 129 |
| 20 | ..... | ..... | 3 | 4 | 2 | 10 | 2 | ..... | ..... | ..... | ..... | 15 | 36 | 14 | 28 | 24 | 15 | 18 | 99 |
| 21 | ..... | ..... | ..... | ..... | 4 | 5 | 1 | ..... | ..... | 2 | 9 | 72 | 93 | 66 | 50 | 9 | 11 | 39 | 175 |
| 22 | ..... | ..... | 1 | 3 | 44 | 30 | 2 | ..... | 1 | ..... | 2 | 15 | 98 | 40 | 54 | 85 | 36 | 16 | 231 |
| 23 | ..... | ..... | 1 | 2 | 25 | 25 | 3 | 3 | ..... | 1 | 5 | 11 | 76 | 22 | 11 | 24 | 10 | 26 | 93 |
| 24 | ..... | ..... | 1 | 6 | 43 | 18 | 2 | ..... | ..... | 1 | 27 | 145 | 243 | 124 | 95 | 78 | 61 | 63 | 421 |
| 25 | ..... | ..... | ..... | 11 | 11 | 19 | 6 | ..... | ..... | 3 | 11 | 49 | 110 | 37 | 29 | 65 | 9 | 9 | 149 |
| Total cases | 1 | 9 | 13 | 48 | 227 | 154 | 58 | 7 | 10 | 116 | 864 | 1671 | 3178 | 874 | 772 | 555 | 268 | 228 | 2697 |
| " deaths. | ..... | ..... | ..... | 1 | 1 | 3 | 5 | 1 | ..... | ..... | 21 | 18 | 50 | 16 | 15 | 8 | 7 | 5 | 51 |

Total cases, 5,875. Total deaths, 101.

According to this table it will be seen that there was only one case in January, 1899, with no deaths; 9 in February, 13 in March, 48 in April and 1 death, 227 in May and 1 death, 154 in June and 3 deaths, 58 in July and 5 deaths, 7 in August and 1 death, 10 in September, 116 in October, 864 in November, with 21 deaths, 1,671 in December with 18 deaths making a total of 3,178 cases with 50 deaths.

In January, 1900, there were 874 cases and 16 deaths, in February, 772 cases and 15 deaths, in March, 555 cases and 8 deaths, in April, 268 cases and 7 deaths, in May, 228 cases and 5 deaths, making a total of 2,697 cases and 51 deaths for the first five months of 1900, or a grand total of 5,875 cases and 101 deaths since the beginning of the year 1899.

By reference to the annual report of the Superintendent of Education for Buffalo, we find that this number represents about one-tenth of the pupils registered in the grammar schools last year. An epidemic which will attack one-tenth of all the school children in a city is certainly worthy of the greatest attention. Not all schools suffered alike, but at times certain schools were so depleted as to give the question a serious aspect from several sources. From the knowledge obtained at the Department of Health, we know that this figure of 5,875 does not represent the entire number of cases of measles which occurred in Buffalo during the period in question. It is only a fractional part, but as we have no means of indication we are unable to give, even approximately, the total. This is owing to the fact that few cases which did not come under a physician's observation were reported, and those that were so reported are an indication that the epidemic contributed a trifle toward the support of the many hundred physicians in this city. A record of many cases was obtained when the children presented themselves for re-admission to school and found it necessary to obtain a proper permit. Hundreds of cases were unattended by physicians, either because of a lack of appreciation of the gravity of the disease, or because they wanted to await the outcome, or for economic considerations.

And right here I might refer to what was undoubtedly the greatest factor in spreading the disease like a prairie fire—aside from other probable conditions which undoubtedly influence epidemics of this nature.

As previously intimated there is a deep-rooted sentiment—often expressed even by physicians—that every person must have measles at some period of life, and that infancy or childhood is the best period. True it is that measles is a disease of childhood, and of all that class of diseases the most prevalent, but it is equally true that the best time to have measles—or any other disease—is never.

Under the influence of this sentiment, it is no wonder, then, that so little precaution is taken, or that we find an almost entire absence of precaution, except where actually enforced.

Children were frequently found attending school, or mingling with others, while members of their families were seriously ill, and it is common knowledge that parents often expose their children who have never had measles, so as to have it over.

Inspectors frequently found children covered with the rash of measles from head to foot running about and freely mingling with others, when they ought to have been carefully tucked in a warm bed, under quarantine.

According to the rules of the Health Department every case of measles must be reported, as well as other contagious or infectious diseases. The quarantine in this instance is not as severe as in scarlet fever or diphtheria and the premises are not placarded. The object of the notification, however, is to obtain a record of the case for statistical protective and preventive purposes. As previously indicated, it is not exact in the statistical branch for the reason that most of the cases which do not come to the notice of physicians are not reported. But there are more cases reported now than ever before, and the experience gained by all interested in the present epidemic is of great value.

Protective and preventive measures are instituted by notifying schools, libraries, etc., of the existence of the disease, so that members of that family may be excluded. The children are not permitted to reënter school until they produce a certificate from the attending physician, countersigned by the Department of Health.

This procedure seems unnecessarily severe at first thought, and has led to many arguments. It is contended that if the child who is ill remains away from school the others ought to be permitted to attend, while others believe that the physician's certificate ought to be sufficient without the formality of approval by the Department which is regarded in many instances as an unnecessary hardship, in that it demands a trip uptown with a possible expenditure of car-fare and the like.

All these steps, however, are the result of experience and have been gradually adopted to improve the system. It frequently happened that in mild cases the physician left a school certificate on making his final call, under the impression that it would not be used until the proper time, or he would give a certificate when the family learned the nature of the disease and told him not to call again, and this certificate would readmit to school, while others, who took the disease later were at home ill. The countersigning by the department, acts as a check, as every case is looked up before approval is given, and a record of each step is kept, so that the department has a complete history of every case and can give definite information to every inquirer. This information is most frequently sought after by school principals and teachers as well as employers.

The question of inconvenience or hardship is not entitled to consideration where the safety of the public is concerned. It is better to inconvenience one family than to expose an entire school.

It is a pleasure to note the intelligent and willing coöperation on the part of public school principals. As their salaries depend upon the attendance they naturally have a pecuniary interest, but so diligent were they in assisting to stamp out the disease that some were actually accused of going to the other extreme. Coöperation between the

School and Health Departments does much toward lessening an outbreak of any contagious disease.

It is not my intention to say anything regarding the treatment, but will leave that for those who discuss this paper. But I desire to say in conclusion that, although it is doubtful if measles, *per se*, is the cause of many deaths, yet during the recent epidemic many cases were reported as ranking with malignant scarlet fever in severity. From this we infer that the complications, which are usually credited with taking the life of the patient, are not always responsible, but it is equally true that if the disease had been taken more seriously there would not be such an outrageous credit to it in the death column.

460 Glenwood Avenue.

---

